# Hyperosmotic stress: *in situ* chromatin phase separation

**DOI:** 10.1080/19491034.2019.1710321

**Published:** 2020-01-10

**Authors:** Ada L. Olins, Travis J. Gould, Logan Boyd, Bettina Sarg, Donald E. Olins

**Affiliations:** a Department of Pharmaceutical Sciences, College of Pharmacy, University of New England, Portland, ME, USA; b Department of Physics & Astronomy, Bates College, Lewiston, ME,USA; c Division of Clinical Biochemistry, Biocenter, Innsbruck Medical University, Innsbruck, Austria

**Keywords:** Histones, chromatin, mitotic chromosomes, osmotic pressure, sucrose, NaCl, dehydration

## Abstract

Dehydration of cells by acute hyperosmotic stress has profound effects upon cell structure and function. Interphase chromatin and mitotic chromosomes collapse (“congelation”). HL-60/S4 cells remain ~100% viable for, at least, 1 hour, exhibiting shrinkage to ~2/3 their original volume, when placed in 300mM sucrose in tissue culture medium. Fixed cells were imaged by immunostaining confocal and STED microscopy. At a “global” structural level (μm), mitotic chromosomes congeal into a residual gel with apparent (phase) separations of Ki67, CTCF, SMC2, RAD21, H1 histones and HMG proteins. At an “intermediate” level (sub-μm), radial distribution analysis of STED images revealed a most probable peak DNA density separation of ~0.16 μm, essentially unchanged by hyperosmotic stress. At a “local” structural level (~1-2 nm), in vivo crosslinking revealed essentially unchanged crosslinked products between H1, HMG and inner histones. Hyperosmotic cellular stress is discussed in terms of concepts of mitotic chromosome structure and liquid-liquid phase separation.

## Introduction

During the entire history of biological evolution, emerging ‘water-based’ single cells and multicellular organisms have had to adapt to multiple dehydration crises. Drought and desiccation, salination by evaporation of water sources, all constituted grave threats to the survival of the soft matter, that we call ‘protoplasm’. The survival of life has required the evolution and preservation (in DNA) of numerous adaptive mechanisms that have created this remarkable cellular homeostasis. This article focuses upon one small feature of these complex adaptations: hyperosmotic effects on the chromatin and chromosomes of living mammalian cells.

Hyperosmotic solutions can be defined as (aqueous) buffers with higher solute concentrations than those of living cells; isosmotic solutions possess solute concentrations comparable to living (mammalian) cells (~280-295 milliOsmolar, ‘mosM’ [1]). Hyperosmotic solutions tend to dehydrate cells; i.e., remove intracellular water through the (semi-permeable) cell membrane. These solutions can be characterized by measurements of ‘osmotic pressure’, the pressure required to counter the flow of water from a cell into the surrounding medium. Mammalian tissue culture media and phosphate buffered saline (PBS) are regarded isosmotic to mammalian cells. 150 mM NaCl is isosmotic and isotonic (i.e., containing comparable levels of cations and anions, as in living cells); whereas 300 mM sucrose is isosmotic (but not ‘isotonic’, since it is not ionic) [1,2]. Sucrose and NaCl do not penetrate into live mammalian cells and therefore, when added to tissue culture media, generate hyperosmotic solutions and dehydrate the cells.

The effects on interphase nuclear structure and functions, obtained by exposing living tissue culture cells to hyperosmotic ‘stress’, have been well documented [3–13]. From the point-of-view of interphase nuclear architecture, it has been clearly demonstrated that hyperosmotic conditions induce increased chromatin heterogeneity (i.e., nuclear regions exhibiting chromatin ‘deficiency’, surrounded by regions with enhanced chromatin compaction); whereas, isosmotic conditions reveal a more homogeneous chromatin distribution [3,4,7,11,12]. Interphase chromatin compaction is a very rapid event, taking less than 20 seconds following exposure to the hyperosmotic medium [11]. Numerous nuclear functions are ‘adversely’ affected by hyperosmotic treatment, including reduced DNA replication and cell division [9,10] and increased apoptosis [9,14]. Supporting these (and other) functional responses are mRNA transcription changes provoked by the hyperosmotic treatment [10,15]. Evidence has been presented that hyperosmotic induction of interphase chromatin condensation is independent of the formation and stability of physiological heterochromatin [12]. However, the effects of hyperosmotic stress on mitotic chromosomes have, apparently, not been reported.

Live mammalian cells incubated for lengthy time periods in hyperosmotic media frequently undergo ‘recovery’, with a return to ‘normal’ cell volume. Phases in this recovery process have been described and analyzed in various mammalian cell types [16,17]. Following hyperosmotic stress, there is a period, called ‘Regulatory Volume Increase’ (RVI); after hypoosmotic stress, a period, called ‘Regulatory Volume Decrease’ (RVD). These recovery periods have been explored in considerable detail [16,17]. Focusing upon hyperosmotic stress, the ‘Acute’ earliest phase (denoted ‘Early events’ in [16]), which may last for ~1 hour, is characterized by rapid cell volume shrinkage, increased internal ionic strength, increased molecular crowding and cell cycle arrest. The subsequent ‘Recovery processes’ [16] last for ~ 1 day and include osmolyte synthesis and cell volume increase, still with cell cycle arrest. Finally, during the following ‘Adaptation’ [16] phase, cells have developed normal cell volumes, transcription, translation and cell cycle. In other words, osmotic stress can be regarded as a reversible perturbation for many cell types.

The present study concentrates upon the ‘Acute’ phase induced with hyperosmotic sucrose (primarily, on HL-60/S4 cells), a period when physicochemical influences likely dominate the changes in chromatin and chromosome architecture. During this Acute period, we have observed condensation of interphase chromatin and mitotic chromosomes (denoted, by us, as ‘congelation’; i.e., to thicken or gel). The dramatic ‘global’ (µm level) structural changes in mitotic chromosomes are discussed in relation to current structural models of mitotic chromosomes [18–20]. Mitotic congelation is accompanied by apparent phase separation of chromatin-associated proteins from the residual chromosome gel. This feature is discussed in relation to current concepts of liquid-liquid phase separation [21–24]. At an ‘intermediate’ (sub-µm) level, employing STED microscopy, DNA image data subjected to Radial Distribution analysis, suggests the persistence of a stable DNA density spacing (~0.16 µm). We also demonstrate that in vivo ‘local’ (nm level) crosslinking of histone and HMG chromatin proteins appear to be unperturbed by acute hyperosmotic stress, when compared to crosslinking in the absence of sucrose. In sum, these data suggest that different ‘levels’ of chromatin structure (i.e., ‘global’ versus ‘intermediate’ versus “local) display differential sensitivity to the perturbation effects of hyperosmotic stress.

## Results

### Acute hyperosmotic stress with sucrose results in shrinkage of viable cells

Exposure of asynchronously growing HL-60/S4 cells to tissue culture medium plus 300 mM sucrose, for up to ~2 hours at 37° C, does not appear to adversely affect cell viability or mitochondrial membrane polarization (depolarization is regarded as enabling apoptosis [25]), as assayed by suitable fluorometric assays (Table 1). In contrast, estimating cell volume using a Beckman Coulter Multisizer 4, revealed that during identical acute hyperosmotic stress, the cells shrink to ~2/3 their original volume (Table 2); slightly less volume shrinkage was observed with 150 mM NaCl. In addition, cell cycle analyses of growing HL-60/S4 cells exposed to 300 mM sucrose revealed only negligible changes in the proportions of G1, S and G2 + M cell phases for at least 2 hours (Table 3). The following immunostaining data were all obtained within the one hour time-frame, when the cells are viable, but somewhat shrunken.

**Table 1.** Viability of HL-60/S4 cells in tissue culture medium + 300 mM sucrose. Each sample was measured in triplicate. The mean values are shown. Viability and Membrane Polarization were measured using separate kits, with the Guava easyCyte single-cell analysis system (EMD Millipore Corp.).

	Viability		Mitochondrial Polarization	
Time in 300 mM Sucrose	% Viable	% Dead	% Polarized	% Depolarized
0	99.5	0.5	94.3	5.7
30 minutes	99.3	0.7	94.1	5.8
1 hour	99.6	0.4	91.7	8.3
2 hours	97.6	2.4	87.6	12.4
3 hours	95.2	4.8	83.0	17.0

**Table 2.** Calculated cell volumes of HL-60/S4. Mean cell diameters were measured on a Beckman Coulter Multisizer 4, employing five separate replicates. Cell volumes were calculated assuming that the cells are spheres. Vol_t_/Vol_0_, ratio of the experimental cell volume at time ‘t’ divided by the control cell volume at time ‘0’ (no sucrose).

CELL VOLUME				
	300 mM Sucrose		150 mM NaCl	
Time in 300 mM Sucrose	Volume (µm)^3^	Vol_t_/Vol_0_	Volume (µm)^3^	Vol_t_/Vol_0_
0	710.5	1.00	711.1	1.00
0.5 hour	433.5	0.61	542.5	0.76
1 hour	433.5	0.61	519.4	0.73

**Table 3.** Flow cytometry of ethanol-fixed, propidium iodide stained cells was performed in a Miltenyi Biotec MacsQuant 10 fluorescence analytical cytometer.

CELL CYCLE ANALYSIS			
Time in 300 mM Sucrose	G1 %	S %	(G2 + M) %
0	45.9	28.9	23.9
15 minutes	41.1	29.4	26.9
30 minutes	40.2	29.9	26.9
60 minutes	41.0	29.2	26.8

### Acute hyperosmotic sucrose stress congeals interphase chromatin and mitotic chromosomes, as viewed by deconvolved confocal immunofluorescent staining

**Figure 1.**
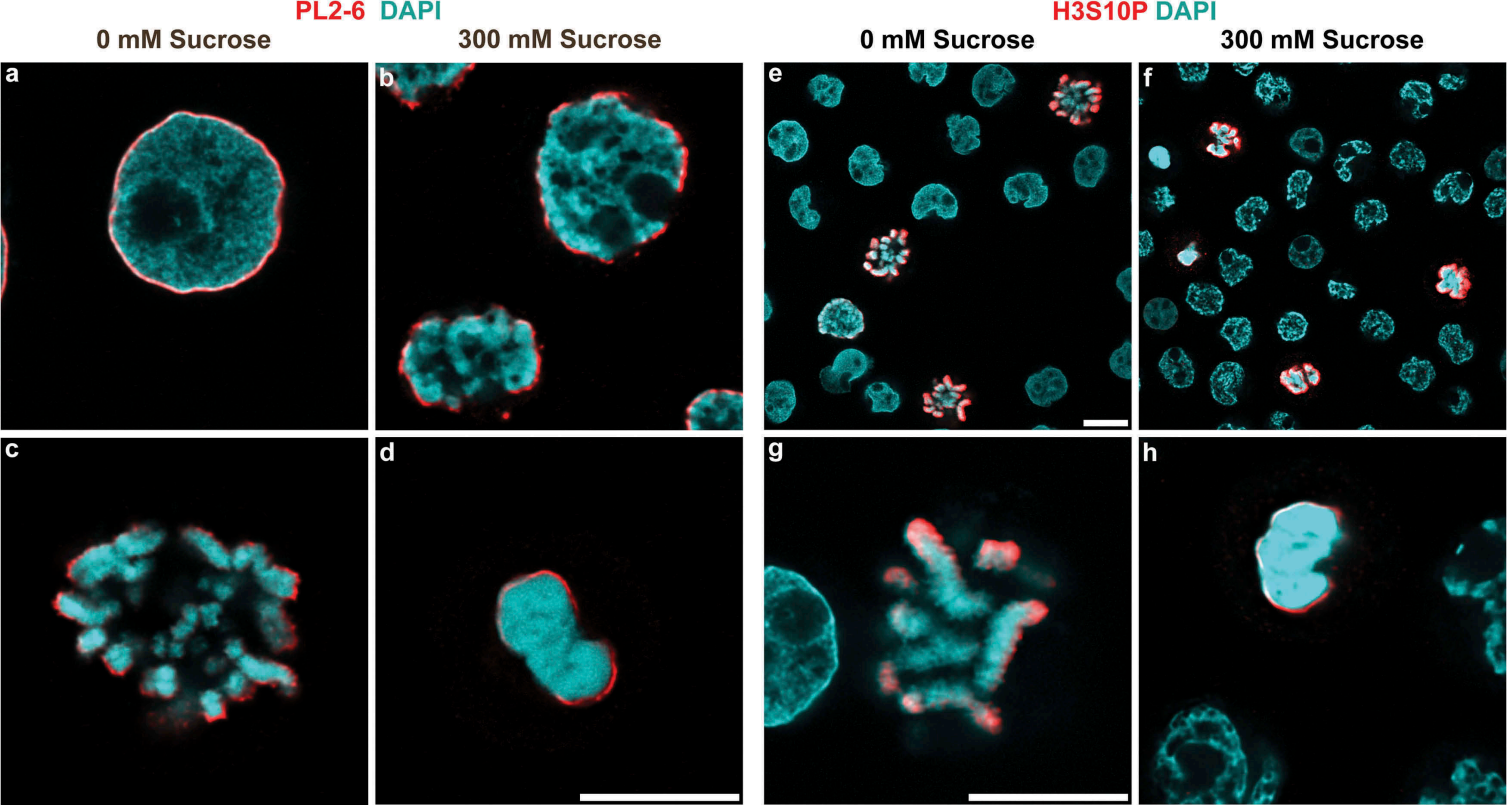
Hyperosmotic sucrose stress causes chromatin congelation. HL-60/S4 Cells incubated ± 300mM sucrose, 37°C, fixed and imaged in a confocal microscope. (a-d) Stained with mAb PL2-6 (anti-epichromatin, red) and DAPI (cyan); (e-h) Stained with Rabbit anti-H3S10p (red) and DAPI (cyan). (a,b) interphase nuclei; (c, d, g, h), mitotic chromosomes; (e, f), low magnification survey of interphase and mitotic cells; (a, c, e, g), 0 mM sucrose; (b, d, f, h), 300 mM sucrose. Magnification bars, 10 µm.

Hyperosmotic sucrose stress for 30 minutes results in significant ‘congelation’ of interphase nuclear chromatin and clustered mitotic chromosomes (Figure 1). Similar interphase chromatin changes (i.e., condensed chromatin granules and fibers interspersed with large ‘chromatin-free’ voids) have been reported earlier [3,11–13]. However, the hyperosmotic ‘gel’ remnants of mitotic chromosome clusters have not been previously described. Figure 1(a) (isosmotic conditions) displays an interphase nucleus possessing a ‘fine’ chromatin (DAPI) meshwork surrounded by ‘epichromatin’ immunostaining (mAb PL2-6, highlighting the surface of chromatin with exposed nucleosome ‘acidic patches’ [26–29]). Figure 1(b) (hyperosmotic 300 mM sucrose) shows an interphase nucleus with coarse chromatin fibers and voids, and epichromatin staining near the nuclear envelope. Figure 1(c) (isosmotic) displays normal mitotic chromosomes with epichromatin staining at the outer surface of the chromosome cluster. Figure 1(d) (300 mM sucrose) presents a congealed amorphous mitotic chromosome cluster surrounded by epichromatin staining. It is clear that treatment of rapidly growing HL-60/S4 cells with hyperosmotic sucrose for 30 min results in dramatic microscopic changes in interphase chromatin and mitotic chromosome architecture. Supplementary Figure S1 displays similar effects of hyperosmotic sucrose stress upon interphase and mitotic U2OS cells.

**Figure 2.**
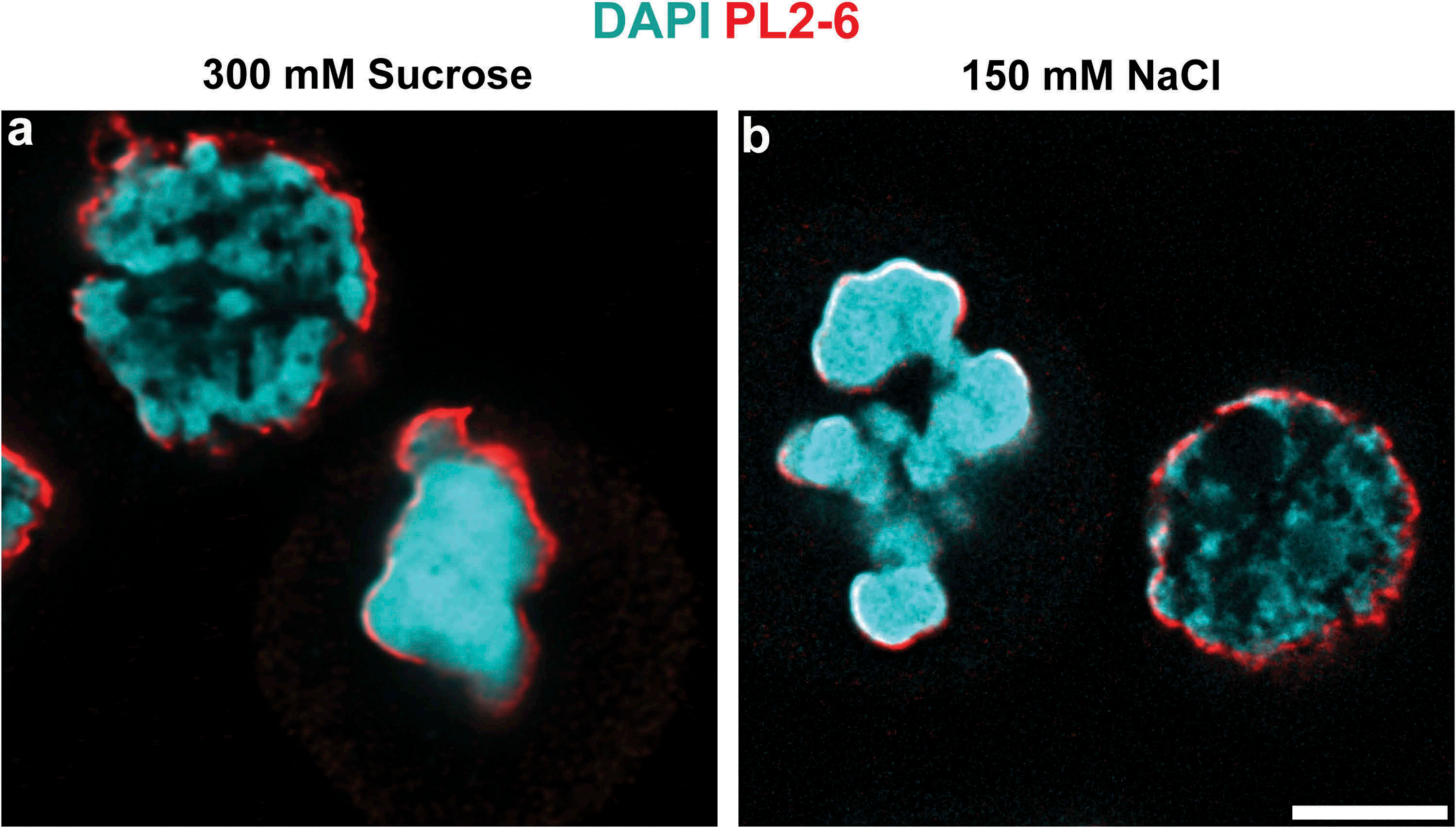
Hyperosmotic NaCl and sucrose stress cause chromatin congelation. HL-60/S4 cells incubated in the presence of 300 mM sucrose (a) or of 150 mM NaCl (b), immunostained with mAb PL2-6 (anti-epichromatin, red) and DAPI (cyan). Each panel contains one interphase nucleus and one cluster of congealed mitotic chromosomes. Magnification bar, 5 µm.

Confirmation of the identity of congealed mitotic chromosomes was obtained by immunostaining of the fixed and permeabilized cells with anti-H3S10p antibody, which has been shown to be specific for mitotic chromosomes in cycling cells [30,31]. Figure 1(e-h) display images of cycling HL-60/S4 cells stained with anti-H3S10p and DAPI. Figure 1(e,g) show cells at isosmotic conditions, demonstrating that only mitotic chromosomes reveal H3S10p staining at the outer surface of the chromosome cluster. Figure 1(f,h) show that congealed mitotic chromosomes maintain the ‘surface’ staining with anti-H3S10p. We have previously shown that anti-epichromatin antibody (PL2-6) staining overlaps and extends slightly outside H3S10p staining [26,28]. It is clear that, as with PL2-6 immunostaining, the H3S10p epitopes remain at (or near) the outer surface of the mitotic chromosome clusters, whether or not they are perturbed by hyperosmotic treatment. Figure 2 compares the chromatin structure disruptive effects of 300 mM sucrose to those of 150 mM NaCl; the immunostained images are virtually indistinguishable. We conclude that ‘acute’ in vivo hyperosmotic treatment of interphase and mitotic cells, whether by sucrose or NaCl, results in a similar perturbation (congelation) of chromatin architecture.

### Super-resolution STED imaging of sucrose and NaCl congealed interphase chromatin and mitotic chromosomes

**Figure 3.**
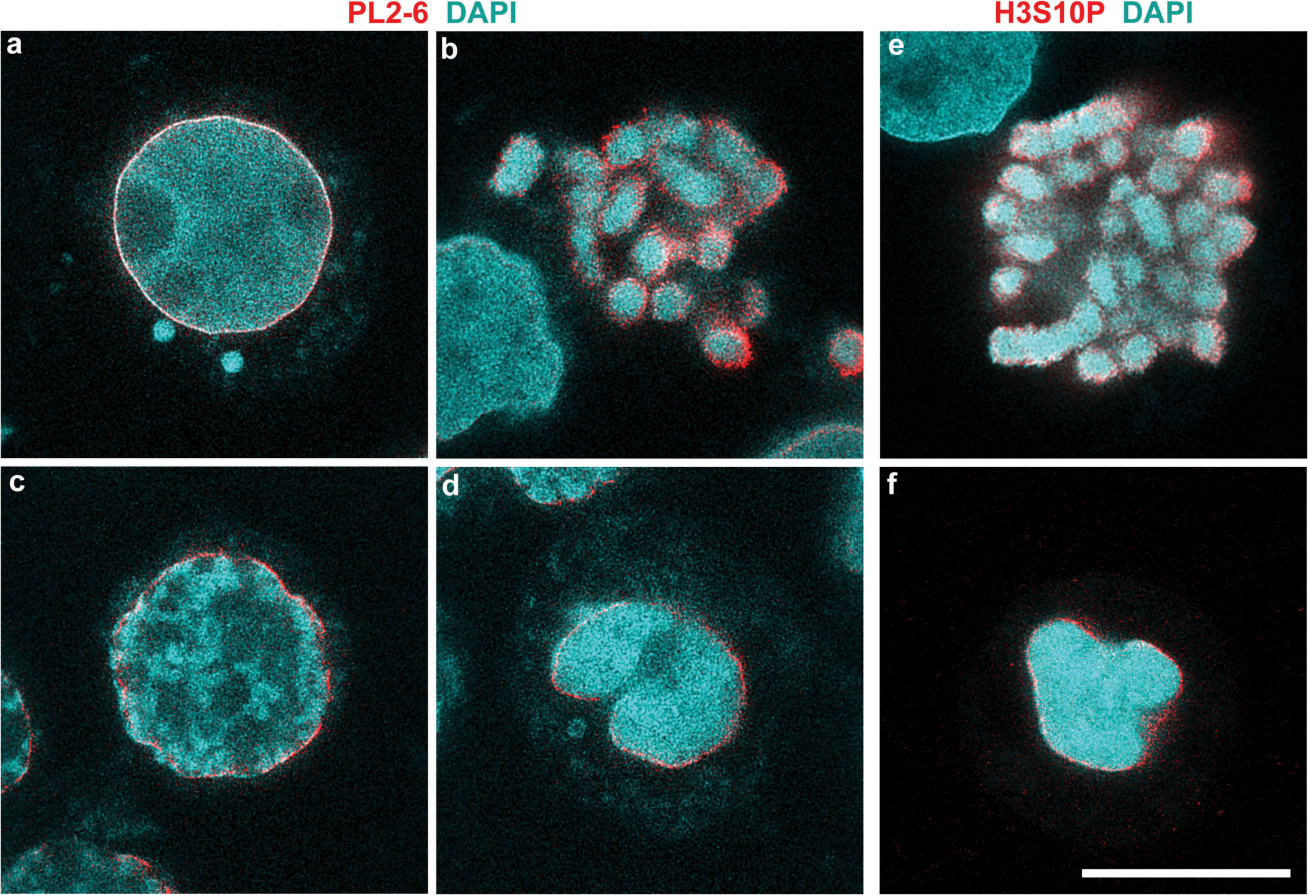
High resolution STED microscopy of HL-60/S4 cells incubated in the absence (a,b,e)/presence (c,d,f) of 300 mM sucrose. (a-d) Stained with mAb PL2-6 (anti-epichromatin, red) and DAPI (cyan); (e,f) Stained with Rabbit anti-H3S10p (red) and DAPI (cyan). Note the ‘sharpness’ of rim staining by mAb PL2-6 and DAPI on the interphase nucleus of panel (a). Magnification bar, 10 µm.

Given the very characteristic morphologies obtained by deconvolved confocal imaging of normal and hyperosmotically perturbed interphase and mitotic chromatin (Figures 1 and 2), we chose to visualize epichromatin and H3S10p immunostaining at higher resolution employing STED imaging. Figure 3(a-d) display PL2-6 and DAPI staining of representative interphase nuclei (Figure 3(a,c)) and mitotic chromosomes (Figure 3(b,d)) at 0 mM (3**a,b,e**, isosmotic) and at 300 mM sucrose (3**c,d,f**, hyperosmotic). Under isosmotic conditions, epichromatin (PL2-6) staining encompasses a thin ‘smooth layer’ (~76 nm thick) in interphase nuclei and appears slightly thicker (~78 nm) and less smooth on mitotic chromosomes, as described earlier [27]. In 300 mM sucrose (3**c,d**), epichromatin staining is still prominent, but less well defined. Our conclusion is that, even under hyperosmotic conditions, surface chromatin ‘exposes’ a high density of nucleosome acidic patches in suitable geometric arrangement to bind mAb PL2-6 with high avidity [27–29]. Figure 3(e) displays anti-H3S10p and DAPI staining of mitotic chromosomes at isosmotic conditions; Figure 3(f), in hyperosmotic conditions. As with PL2-6, the anti-H3S10p immunostaining is not smooth on the surface of isosmotic mitotic chromosomes, with the epitope clearly exposed under both isosmotic and hyperosmotic conditions.

**Figure 4.**
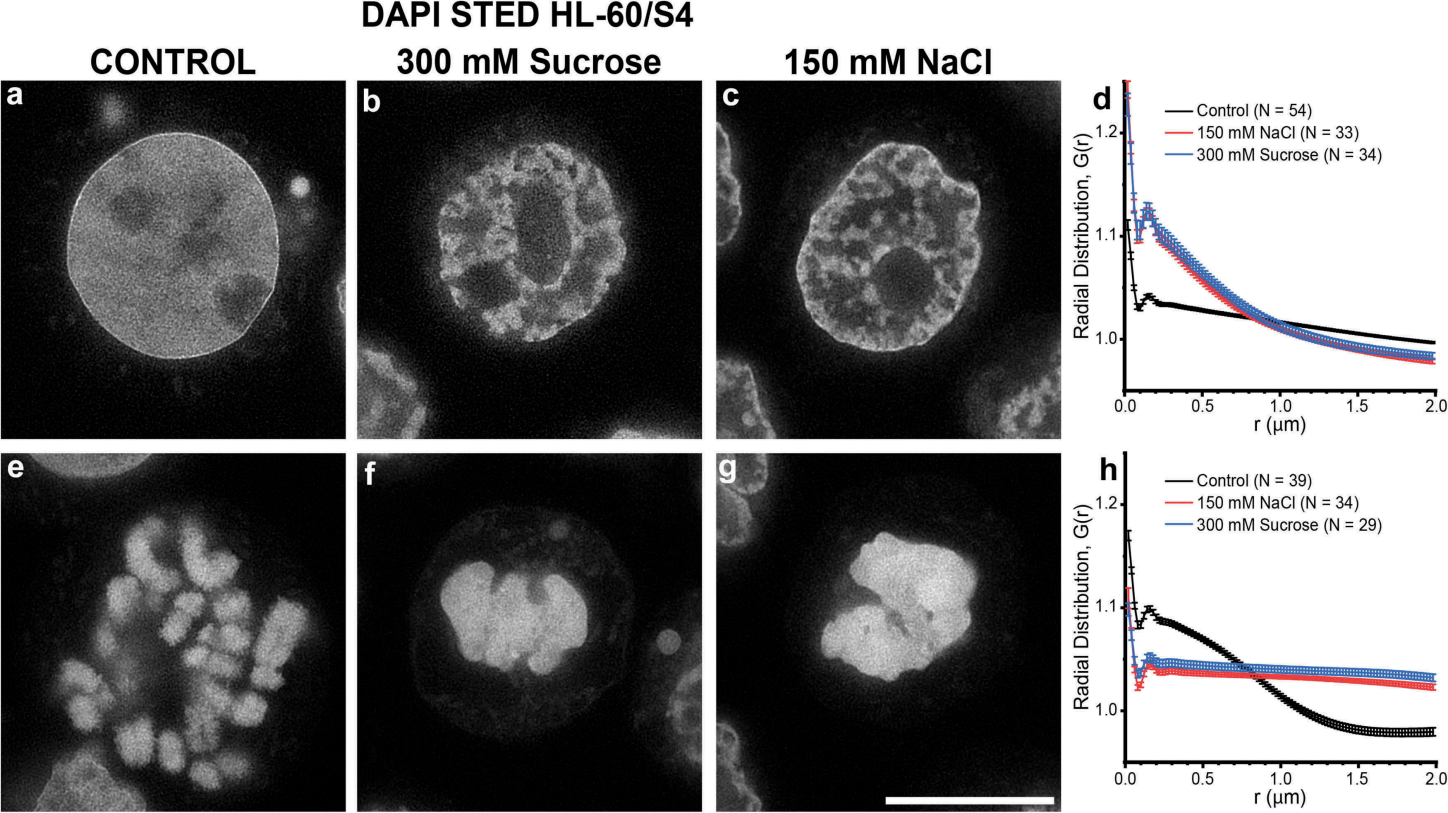
High resolution STED microscopy of HL-60/S4 cells incubated in the absence (a,e)/presence (b,f) of 300 mM sucrose or (c,g) 150 mM NaCl, stained only with DAPI. (a,b,c), interphase nuclei; (e,f,g), mitotic chromosomes. (a,e), Control (0 mM sucrose); (b,f) 300 mM sucrose; (c,g), 150 mM NaCl. DAPI staining is shown in an inverse gray scale, where ‘white’ is the most intense DAPI staining. Note the ‘sharpness’ of rim (‘epichromatin’) staining by DAPI on the Control interphase nucleus (a). Magnification bar, 10 µm. (d,h), graphs of the Radial Distribution function (density-density pair correlation) ‘G(r)’ for DAPI staining of interphase nuclei (a,b,c) or mitotic chromosomes (e,f,g). G(r) values > 1 indicate that the ‘density’ signal is greater than expected for a uniform randomly distributed signal; G(r) values < 1, signal less than uniform. N, the number of nuclei (or congealed chromosome clusters) measured for each profile.

We wanted to analyze DNA substructure at a higher resolution by examining DAPI staining using STED microscopy. Figure 4(a-c) compares STED images of HCHO-fixed, permeabilized and DAPI stained interphase cells (with or without 30 minutes incubation in 300 mM sucrose or 150 mM NaCl); Figure 4(e-g), compares STED images of mitotic cells exposed to the same conditions as the top row. These DAPI stained STED images demonstrate a very clear and similar ‘fibro-granular’ chromatin substructure in all of the conditions shown. The Radial distribution function (also known as ‘density-density pair correlation’) was calculated for interphase nuclear chromatin (Figure 4(d)) and for mitotic chromosomes (Figure 4(h)). For all these graphs, a single small peak at ~0.16 µm, was observed. Table 4 presentsthe mean radial distribution function values (± standard error) for each group of ‘N’ nuclei or chromosomes as shown in Figure 4, calculated from the 2D DAPI images (see **Materials and Methods**). It is clear that (despite the considerable ‘global’ chromatin structural perturbations, comparing interphase nuclei and mitotic chromosomes of sucrose or NaCl treated live cells) the single small peak values are all very similar in ‘r(µm)’. These calculated peaks represent the probability of finding a DNA-dense ‘particle’ at a specific distance (µm) from any reference DNA-dense particle. This apparent constancy of peak distances suggests that congelation brings together DNA-dense chromatin regions that already possess close-packed ‘particulate’ structures, which (under these experimental conditions) do not pack any closer. At larger distances, some interesting differences between the samples provokes speculation: 1) For mitotic chromosomes (Figure 4(h)), the control isosmotic conditions display a radial distribution with a ‘steep slope’ that levels off at ~1.25 µm, possibly correlating with the width of chromosome arms. After sucrose or NaCl treatment, the congealed chromosome radial distribution functions are ‘flatter’, perhaps reflecting a more uniform chromatin distribution. 2) For interphase nuclei (Figure 4(d)), control isosmotic conditions appear to promote a near uniform and random distribution of chromatin fibers at radial separation distances larger than 0.16 µm. However, after hyperosmotic stress, interphase nuclei display heterogeneous chromatin strands with multiple thicknesses less than ~1 µm. Ironically, the congealed mitotic chromosome radial distribution plots most resemble the plots of the ‘unperturbed’ interphase nuclei.

**Table 4.** Radial Distribution Analysis (Density-Density Pair Correlation) of STED images of DAPI (DNA) stain distribution in control (or congealed) interphase nuclei or mitotic chromosomes, see Figure 4. Mean values were calculated from N objects, ranging from N = 29–54.

Radial Distribution (Density-Density Pair Correlation)				
	Interphase		Mitotic	
	Peak (µm)	± (µm)	Peak (µm)	± (µm)
Control	0.164	0.042	0.157	0.022
300 mM Suc	0.155	0.042	0.17	0.041
150 mM NaCl	0.156	0.043	0.171	0.04

### Ki67 segregates from hyperosmotic sucrose congealed mitotic chromosomes

A recent study [32] demonstrates that the nuclear protein Ki67 acts as a (+) charged surfactant, coating and separating (by repulsion) the mitotic chromosomes. The chromosome repulsion activity arises from the C-terminal leucine-arginine (LR) rich domain. Within interphase nuclei, Ki67 is also important in establishing the boundary between nucleoli and surrounding heterochromatin; for recent discussions of the multiple roles of Ki67, see [33,34]. Disruption of the interaction between Ki67 and mitotic chromosome arms, employing siRNA [32,35] or auxin-induced ‘degron’ degradation [33] of Ki67, results in a collapse of mitotic chromosomes.

**Figure 5.**
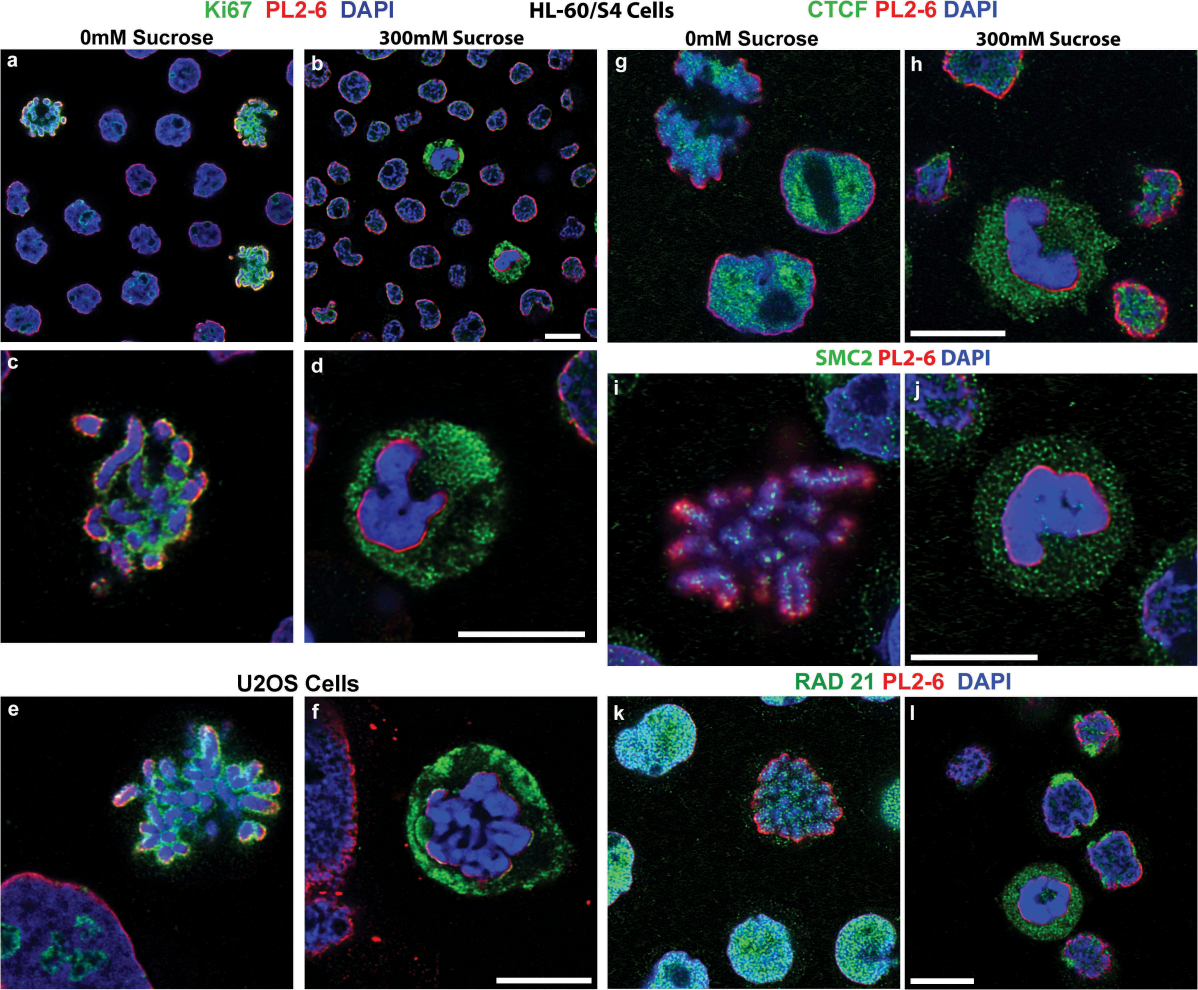
Select nuclear proteins separate from hyperosmotic sucrose congealed mitotic chromatin. Rabbit antibodies against Ki67, CTCF, SMC2 and RAD21 (all green), and counterstained with PL2-6 (red) and DAPI (blue). All panels, except (e,f), show images of HL-60/S4. (a-d) Stained with anti-Ki67. (a,b), HL-60/S4 low magnification, mixed interphase and mitotic cells; (c,d), high magnification, mitotic chromosomes. (e,f) U2OS cells stained with Ki67. (a,c,e,g,i,k), 0 mM sucrose; (b,d,f,h,j,l), 300 mM sucrose. Note the Ki67 staining surrounding nucleoli of 0 mM sucrose interphase nuclei, which are absent from 300 mM sucrose interphase nuclei. (g,h), anti-CTCF. (i,j), anti-SMC2. (k,l), anti-RAD21. The upper left corner of (g) contains a cluster of mitotic chromosomes stained with anti-CTCF. Magnification bars, 10 µm.

We wanted to explore whether the hyperosmotic sucrose stress-induced congelation of mitotic chromosomes influences the distribution of Ki67 in separating the mitotic chromosome arms. Figure 5(a,c) shows that growing HL-60/S4 cells from isosmotic tissue culture medium exhibit mitotic chromosomes with Ki67 interspersed between chromosome arms; Figure 5(b,d) demonstrates that 30 min in medium+300 mM sucrose results in a collapse (congelation) of mitotic chromosomes, with Ki67 segregated from the congealed chromosomes. Also visible, Ki67 no longer ‘coats’ interphase nucleoli in 300 mM sucrose. Figure 5(e,f) presents a similar segregation of Ki67 from congealed mitotic chromosomes in hyperosmotically stressed U2OS cells. Chromosome congelation and Ki67 exclusion are sucrose concentration-dependent, with increased effects seen by STED imaging, comparing 0, 100 and 200 mM sucrose (Supplementary Figure S2). However, these experiments do not establish whether sucrose-induced congelation causes Ki67 segregation.

### Other chromosome structural proteins are excluded from congealed mitotic chromosomes after treatment with hyperosmotic sucrose

Higher-order structure of interphase chromatin and mitotic chromosomes involves proteins that define gene functional boundaries and act to generate and stabilize folded chromatin loops [20,35–37]. Excellent antibodies are available for immunostaining several of these key proteins involved in chromatin looping (e.g., CTCF, SMC2 and RAD21). CTCF acts as a ‘barrier’ between different ‘cis’ genetic regions, complexing with condensin components (including RAD21) to stabilize interphase chromatin loops. Figure 5(g,h) illustrate that after a 30 min treatment with hyperosmotic (300 mM) sucrose), CTCF appears to be segregated from congealed mitotic chromosomes. SMC2, a component of condensin I, involved in mitotic chromosome loop formation, also appears to be ‘excluded’ from congealed mitotic chromosomes (Figure 5(i,j)). Likewise, after treatment with 300 mM sucrose, RAD21 (Figure 5(k,l)) reveals displacement into the cytoplasm beyond the ‘epichromatin’ region of congealed chromosomes; whereas, interphase RAD21 accumulates at the epichromatin region, co-immunostaining with mAb PL2-6. These images strongly support that key chromatin-associated proteins involved with establishing loop domains and normal mitotic chromosome organization are segregated from the congealed mitotic chromosomes.

**Figure 6.**
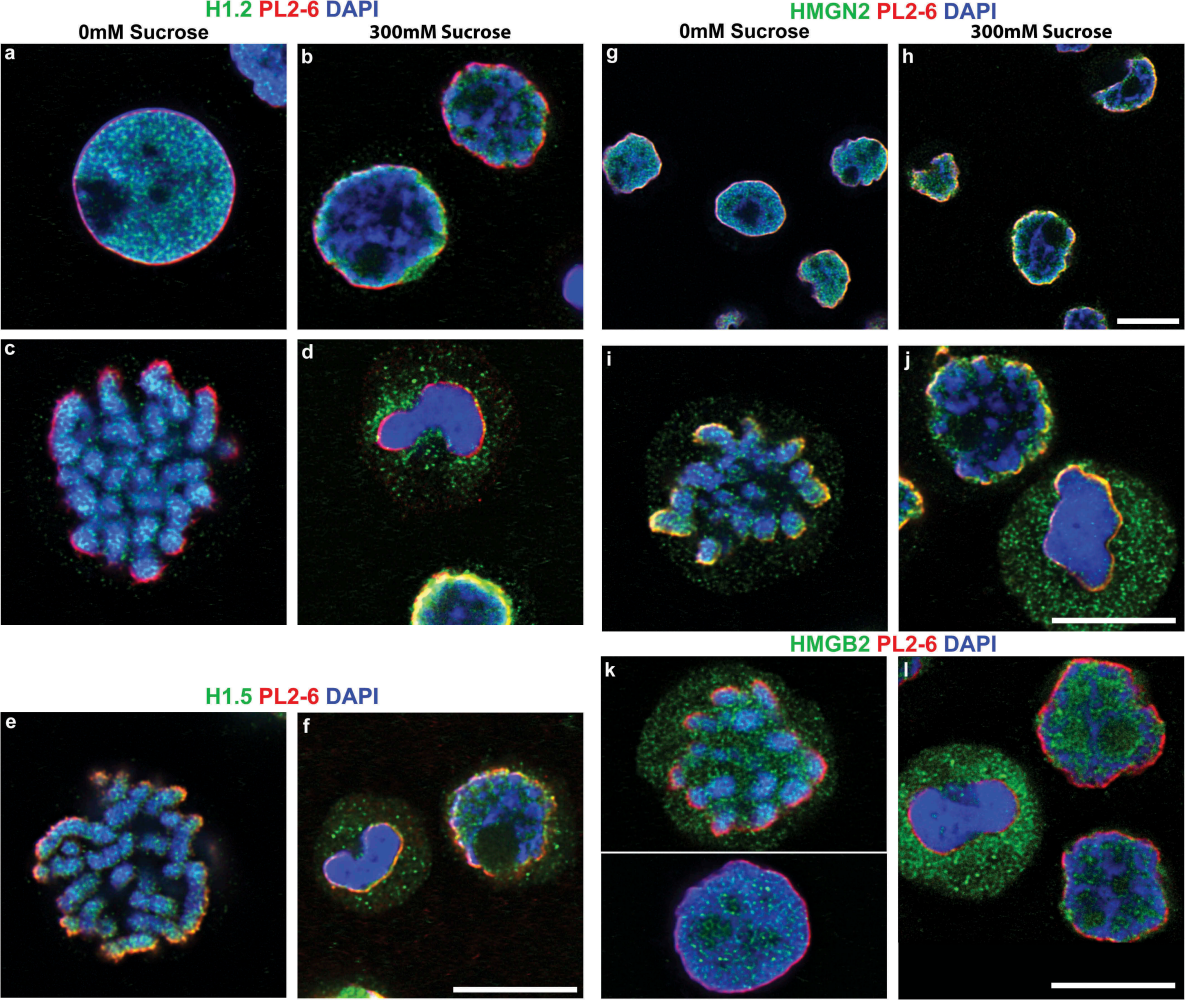
Select chromatin proteins in hyperosmotic sucrose incubated HL-60/S4 Cells. Confocal immunostaining in the absence/presence of 300 mM sucrose, stained with rabbit antibodies against H1.2, H1.5, HMGN2 and HMGB2 (all green), and counterstained with PL2-6 (red) and DAPI (blue). (a-d) stained with anti-H1.2; (e,f), anti-H1.5; (g-j), anti-HMGN2; (k,l), anti-HMGB2, (a,c,e,g,i,k), 0 mM sucrose; (b,d,f,h,j,l), 300 mM sucrose. Magnification bars, 10 µm.

At a more fundamental level of chromatin structure, histone H1 and HMG proteins also exhibit perturbations in localization within hyperosmotically-stressed mitotic chromosomes. Fixed and permeabilized interphase nuclei and mitotic chromosomes display a punctate ‘chromomeric’ immunostaining pattern in 0 mM sucrose (Figure 6(a,c,e)), when reacted with anti-H1 antibodies [for a succinct review of current knowledge concerning H1 and the ‘chromomere’ concept, see [28]]. The punctate staining pattern of mitotic chromosome arms, employing anti-H1.2 and anti-H1.5, largely disappears from the congealed mitotic chromosomes (Figure 6(d,f)) and displays H1 epitopes in the mitotic cell cytoplasm. On the other hand, anti-H1 staining of interphase chromatin appears to migrate with the chromatin toward the nuclear envelope, when treated with 300 mM sucrose (Figure 6(b)). The HMG non-histone proteins constitute a family of proteins (in lesser amounts than H1 histones) which bind to nucleosomes and modulate chromatin higher-order structure and functions [38–40]. Figure 6 presents immunostaining data for two HMG proteins (HMGN2 and HMGB2), illustrating the patterns in 0 mM and in 300 mM sucrose. For these HMG antigens, the results are quite similar to those of the H1 antigens; namely, that a punctate chromomeric pattern is seen within interphase and mitotic chromatin in 0 mM sucrose (Figure 6(g,i,k)), with the staining absent from congealed mitotic chromosomes in 300 mM sucrose (Figure 6(h,j,l)). Surveying many such immunostained cells, it is clear that the hyperosmotically congealed mitotic chromosomes seldom exhibit staining by the tested anti-H1 and anti-HMG antibodies, whose epitopes shift to outside the epichromatin boundary; whereas, congealed interphase nuclei generally show some retained H1 and HMG antibody staining, colocalizing with the redistributed chromatin within the epichromatin boundary.

### In vivo protein crosslinking with cell permeant DSS, comparing isosmotic and hyperosmotic conditions, reveals a surprising ‘constancy’ of cross-linked acid-extracted products

The immunostained redistribution of H1 and HMG proteins outside the epichromatin (PL2-6) ‘boundary’ (i.e., beyond congealed mitotic chromatin), following hyperosmotic sucrose stress (described above), suggests that these proteins have ‘separated’ from their binding partners. We wanted to test this idea by performing in vivo protein crosslinking.

**Figure 7.**
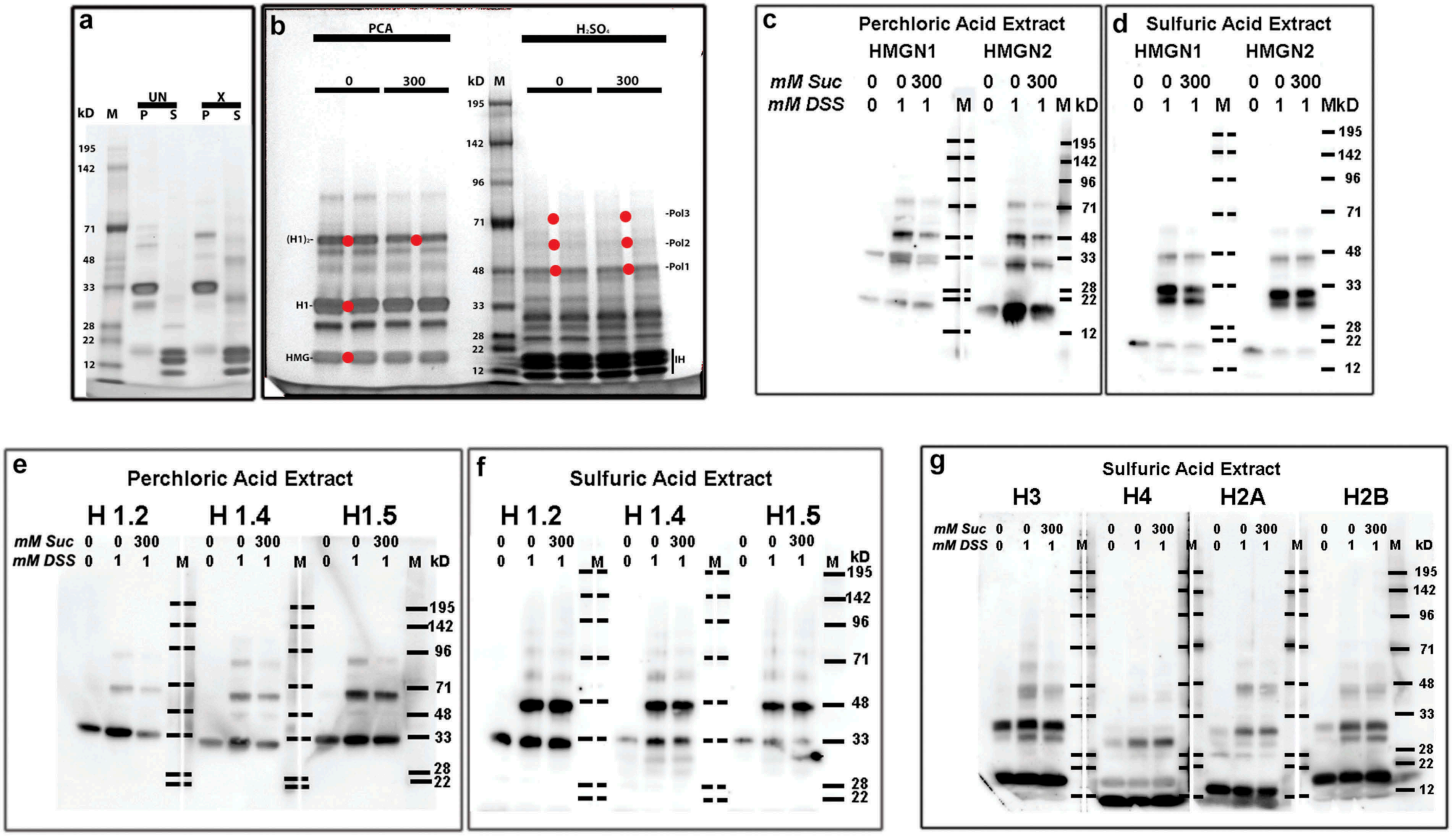
DSS cross-linking of acid extractable chromatin proteins. (a), Coomassie blue (InstantBlue) stained 4-12% acrylamide gradient SDS-PAGE of PCA (p) and H_2_SO_4_ (s) extracts from HL-60/S4 cells crosslinked (or not) with 1 mM DSS in PBS ± 300 mM sucrose. Comparison of uncrosslinked (UN) or crosslinked (x) extracts. Molecular weight markers (m) are aligned with their assigned weights (kD). Note the presumptive H1 monomer (MW ~33 kD) and the presumptive H1 dimers following DSS crosslinking (MW ~68 kD). (b), Comparison of acid extracts from HL-60/S4 cells crosslinked with 1 mM DSS in PBS (pH 8) ± 300 mM sucrose. Each sample was run in duplicate. The red dots placed between duplicate bands indicate the adjacent gel regions that were excised for mass spectroscopy: for 0 mM sucrose [HMG, H1 and (H1)_2_]; for 300 mM sucrose [(H1)_2_, Pol1, Pol2 and Pol3]. ‘Pol’ bands represent presumptive ‘polymers’ of the histones and/or HMG proteins (see Supplementary Table S1). IH, inner histone (H3, H4, H2A, H2B) region. Note that, for panel (b), the DSS crosslinked products show considerable similarity, ± exposure to hyperosmotic sucrose. (c,d) Immunoblots of anti-HMGN1 and anti-HMGN2 reacted with PVDF membranes from 4-12% SDS-PAGE of PCA (c) or H_2_SO_4_ (d) extracts of HL-60/S4 cells crosslinked (or not) with 1 mM DSS in PBS ± 300 mM sucrose. Indicated at the top of each lane are the mM sucrose and the mM DSS employed in that preparation. Note the presumptive HMG monomers (MW ~20 kD) and the presumptive DSS crosslinked products (MW ~33 and ~48 kD) within the PCA (c) and H_2_SO_4_ (d) extracts. Also note that, for panels (c) and (d), these DSS crosslinked products show considerable similarity, ± exposure to hyperosmotic sucrose. Crosslinked products do not appear in the uncrosslinked extracts, except for HMGN1, which showed a weak band at ~33 kD in the PCA extract. (e,f) Immunoblots of anti-H1 antibodies reacted with PCA (e) or H_2_SO_4_ (f) extracts of HL-60/S4 cells. Note that for each histone H1, the DSS crosslinked products show considerable similarity, ± exposure to hyperosmotic sucrose. (g) Immunoblots of anti-inner histone (H3, H4, H2A and H2B) antibodies reacted with H_2_SO_4_ extracts of HL-60/S4 cells. Note that for each inner histone, the DSS crosslinked products show considerable similarity, ± exposure to hyperosmotic sucrose.

Disuccinimidyl suberate (DSS) is a cell permeable, homobifunctional chemical cross-linker with reactivity to primary amines, as in the lysine sidechain. The crosslink is 1.14 nanometers in length and very stable, even in strong acids. HMG proteins and H1 histones can be efficiently extracted by incubating whole cells in 5% perchloric acid (PCA); inner histones (H3, H4, H2A and H2B) are extractable in 0.4 N sulfuric acid (H_2_SO_4_). Following a series of DSS titration experiments, we settled upon a protocol (see, **Methods and materials**), involving addition of live HL-60/S4 cells (previously incubated in tissue culture medium ± 300 mM sucrose for 30 min) to PBS (pH 8, ± 300 mM sucrose) with a final concentration of 1 mM DSS, ~17% DMSO for 5 min, followed by quenching unreacted DSS with 1 M Tris buffer (pH 7.5). Subsequent extractions with PCA, followed by H_2_SO_4_, yielded two fractions, one enriched with HMG and H1; the other, enriched with inner histones. (5 min at 5 mM DSS in vivo rendered HMG and H1 completely nonextractable with PCA). The PCA and H_2_SO_4_ extracts were electrophoresed in 4–12% acrylamide gradient SDS gels, stained with Coomassie blue (InstantBlue) or transferred to PVDF membranes for immunoblotting. Figure 7(a,b) presents a composite of two separate gels stained with InstantBlue. Figure 7(a) compares PCA (‘P’) and H_2_SO_4_ (‘S’) extracts from uncrosslinked (‘UN’, 0 mM DSS) and crosslinked (‘X’, 1 mM DSS) live HL-60/S4 cells. Figure 7(b) compares PCA and H_2_SO_4_ extracts from cells that were crosslinked with 1 mM DSS (5 min) during treatment with 0 or 300 mM sucrose (30 min). Each extract sample was loaded in duplicate. The names assigned to each band should be regarded as ‘convenience names’. The red dots in Figure 7(b) indicate where gel slices were obtained for mass spectroscopic (MS) analyses. The most striking observation from these stained gels is that there are very few differences between the crosslinked band patterns, comparing 0 to 300 mM sucrose, for both the PCA and H_2_SO_4_ extracts, despite the considerable ‘global’ chromatin perturbations, described earlier, resulting from hyperosmotic stress. Based upon parallel immunoblotting experiments and MS analyses, we can say that, for both UN and X, the major PCA band at ~33-35 kD includes (monomer) H1 and the minor band at ~17-18 kD contains (monomer) HMG proteins. In the PCA extract of ‘X’, the moderately staining band at ~68 kD is a strong candidate for H1 dimers. It is also apparent that under our chosen conditions of DSS crosslinking, most of the HMGs, H1s and inner histones are not crosslinked.

Immunoblotting experiments support the observation from stained gels of minimal differences between acid-extractable protein products, following crosslinking with DSS in 0 or 300 mM sucrose. Figure 7(c,d) present immunoblot comparisons of extracted HMGN1 and HMGN2. In Figure 7(c), the PCA extract patterns resulting from crosslinking with 1 mM DSS in either 0 or 300 mM sucrose are substantially similar to each other and clearly different from the uncrosslinked extract; PCA extracts are devoid of inner histones (not shown). Uncrosslinked HMGs are migrating at ~20 kD. Especially interesting is the ~48 kD band seen in DSS treated PCA extracts, possibly due to a bond between H1 and an HMGN. The band at ~33 kD is puzzling but could represent an HMGN dimer. There is also a weak band at ~72 kD, confined to the crosslinked extracts. Figure 7(d) displays H_2_SO_4_ extracts blotted with anti-HMGN1 and N2. A low MW band (~20 kD), presumably uncrosslinked HMGN1 and N2, diminishes in intensity in the DSS crosslinked samples. An intense doublet in crosslinked samples between ~30-33 kD suggests additional products joining an HMGN with an inner histone. A new band appears at ~45kD. Figure 7(e,f) presents a comparison of PCA and H_2_SO_4_ extracts from uncrosslinked cells and cells crosslinked with 1 mM DSS in either 0 or 300 mM sucrose, blotted with anti-H1.2, H1.4 and H1.5. HL-60/S4 cells primarily possess only these three H1s [41]. Figure 7(e) displays the PCA extracts. Candidate band putative identities: 1) uncrosslinked H1s migrate at ~ 33 kD. 2) DSS crosslinked H1s migrate at ~65 (H1 dimer?) and ~90 kD (H1 trimer?), with a very weak band at ~48 kD. The presumptive H1 dimer and trimer are not observed in the uncrosslinked extract. Consistent with the InstantBlue stained geIs (Figure 7(a,b)), it is clear that a considerable proportion of the H1s are not crosslinked by the 1 mM DSS. A similar immunoblot comparison of H1.2, H1.4 and H1.5 within the H_2_SO_4_ extracts is presented in Figure 7(f). There are several interesting comparisons between Figure 7(e,f): 1) a substantial amount of uncrosslinked H1s are present in the H_2_SO_4_ extracts. 2) the ~48 kD band is now stronger than the ~65 kD band in Figure 7(f), suggesting a crosslink between H1 and an inner histone. 3) very weak H1 bands are detected in Figure 7(f), at ~65, 75 and 90 kD, with the blot patterns essentially unchanged, comparing crosslinking in either 0 or 300 mM sucrose. Finally, Figure 7(g) displays H_2_SO_4_ extracts immunoblotted with anti-H3, H4, H2A, and H2B. Although the exact composition of most bands is lacking, there is a general resemblance of crosslinked products for each antibody with or without sucrose. In summary, immunoblotting for HMG, H1 and inner histone proteins reveals very few obvious differences in crosslinked products, comparing extracts from cells treated with 1 mM DSS in medium ± added 300 mM sucrose; whereas, there are significant differences, when DSS crosslinked extracts are compared to uncrosslinked cell extracts.

In an attempt to provide additional characterization of the DSS crosslinked products, generating hypotheses of in vivo proximities, we performed mass spectrometry (MS) on bands excised from Coomassie blue-stained gels (Figure 7(b)). A compilation of ‘significantly probable’ nuclear proteins (emphasizing HMG, H1 and inner histones and excluding contaminants; e.g., cytokeratins) is presented in Supplementary Table S1. For each column of Table S1, the ‘convenience name’ of a band is indicated. As has been repeatedly observed, the apparent molecular weights (MW) of ‘monomer’ HMG, H1 and inner histones measured by SDS-PAGE is greater than the calculated MW (from amino acid composition), due to the high number of basic residues which partially cancel out the (-) charges from bound SDS. Supplementary Table S1 also indicates (by closed and open dots) the correspondence of apparent MW and protein identity derived from immunoblot analyses, as in Figure 7(c-g). Generally, there is good agreement between MS and antibody binding, when a specific antibody was available and there were enough signature peptides for the MS assignment. It is of historical interest that the first indication of H1 proximity to other histones within intact nuclei came from glutaraldehyde-fixation, followed by H_2_SO_4_ extraction and analysis of electrophoresis bands by amino acid analysis, detected as H1 containing polymer proteins [42]. In summary, both immunoblotting and MS data indicate that many in vivo DSS crosslinked acid-extracted products have apparently similar electrophoretic mobility and protein composition, comparing cells that were crosslinked in medium containing 0 or 300 mM sucrose.

## Discussion

The present investigation explores the effects of acute hyperosmotic stress upon chromatin architecture at several different dimensional scales: ‘global’ (µm), employing immunostaining confocal and STED microscopy; ‘intermediate’ (100–200 nm), analyzing STED microscopy radial distribution measurements; ‘local’ (nm), employing in vivo protein crosslinking. Different degrees of change and constancy are observed, comparing these three dimensional scales.

At the global level, exposure of live HL-60/S4 cells to 300 mM sucrose in tissue culture medium (i.e., ~twice isosmotic conditions) for 30 min results in coarse heterogeneous interphase chromatin condensation, as reported by others [3,4,7,11–13]. In addition (and apparently unreported), mitotic chromosomes collapse. Interphase and mitotic events (denoted by us, ‘congelation’) occur very rapidly [interphase condensation in <20 sec [11]] after exposure to hyperosmotic conditions and are accompanied by apparent rearrangements and segregations of some chromatin-associated proteins. The congealed mitotic chromosomes as viewed by deconvolved confocal and by STED imaging appear largely amorphous with close-packed chromatin fibers. Key proteins involved with interphase and mitotic chromatin higher-order structure (i.e., Ki67, CTCF, SMC2 and RAD21) appear to be dislodged from their ‘normal’ locations. Congealed mitotic chromosomes also exhibit repositioned HMG and histone H1 proteins. Thus, hyperosmotic stress induces massive ‘global’ chromatin and chromosome perturbations.

At the intermediate scale, STED imaging of DAPI (DNA) staining, combined with radial distribution function analysis (also known as ‘density-density pair correlation’) identifies a ‘peak’ at ~0.16 µm, which is surprisingly constant, comparing 0 and 300 mM sucrose, and 150 mM NaCl. This ‘density-density separation distance’, present in the three osmolarity states studied here provokes speculation about an underlying stable chromatin higher-order structure, even after congelation. It is possible that this peak separation value represents average distances between the centers of ‘chromomeres’ [28], the presumptive HCHO-fixed counterpart of ‘compact chromatin domains’ (~0.20 µm diameter) in vivo [43], particulate structures rich in nucleosomal DNA.

At the ‘local’ scale, in vivo protein crosslinking employing 1 mM DSS (disuccinimidyl suberate, which crosslinks lysine amino sidechains with ~1.14 nm separation) reveals essentially identical patterns of crosslinked HMG, H1 and inner histone proteins, whether the DSS crosslinking is performed in 0 or 300 mM sucrose. The acid-extracted products of DSS crosslinking were examined by SDS-PAGE, followed by Coomassie Blue staining, imaging and bands excision for mass spectroscopic (MS) identification. In addition, unstained gels were transferred to PVDF membranes for immunoblotting (IB). Combining the gel imaging, MS and IB results permits several general conclusions: 1) Some bands are present after crosslinking, that are not observed in uncrosslinked extracts. 2) At the crosslinking conditions employed (1 mM DSS for 5 min), a considerable fraction of HMG, H1 and inner histones are not crosslinked. 3) The crosslinked products obtained after DSS treatment in PBS (pH 8) plus 0 or 300 mM sucrose looked virtually identical. 4) Putative crosslinked band compositions are suggested when HMGs and H1s (which are normally extracted with 5% PCA) were identified in H_2_SO_4_ extracts, exhibiting a higher molecular weight than the monomer proteins. For example, ~48 kD bands in H_2_SO_4_ extracts show the presence of HMGN1, HNGN2, H1.2, H1.4, H1.5, H3, H2A and H2B. These bands likely represent co-migration of numerous crosslinked products. Their analysis could be useful for determining proximities in vivo. The needed experiments probably will involve immunoprecipitation and MS cleavable crosslinkers.

What are the mechanisms underlying interphase and mitotic chromatin congelation during acute hyperosmotic stress? As described earlier, acute hyperosmotic stress of HL-60/S4 cells results in shrinkage of the still viable cells (Tables 1 and 2). In growth medium+300 mM sucrose the mean peak cell volume shrinks to ~61% of the cell volume in isosmotic medium (Table 2), due to the dehydration effect. [Similar measurements of HL-60/S4 cells in medium ± 150 mM NaCl yielded slightly less shrinkage (~73-76%, Table 2), possibly due to the incomplete dissociation of NaCl in aqueous solution [1]]. Besides the increased ‘crowding effect’, we would expect that intracellular ionic and nonionic solutes would increase in concentration during sucrose-induced dehydration, possibly increasing the internal ionic strength to ~500 mM (i.e., ~300 mM x 1/0.61) and weakening macromolecular interactions that have a significant electrostatic component. It is very well known that ~300 mM NaCl extracts HMG proteins from isolated chromatin, ~600 mM NaCl extracts H1 proteins and 1–2 M NaCl extracts inner histones [44]. Thus, increased internal ionic strength could mobilize these chromatin-associated proteins, changing their locations and establishing new interactions with the many redundant sites on chromatin, apparently yielding unchanged crosslinked products. However, it is also possible that, due to dehydration, the shrinking cell volume leads to higher concentrations of chromatin-associated proteins (including HMG, H1 and inner histone proteins) resulting in a ‘tighter’ binding to chromatin. This could be one other explanation for the similarity of crosslinked products, comparing isosmotic and hyperosmotic conditions.

Are hyperosmotically congealed mitotic chromosomes related to a ‘liquid droplet’ model of chromatin? Within the past few years, there has been an ‘explosion’ of experimentation, analyses and thermodynamic explanations of ‘liquid-liquid phase separation, (LLPS)’ within the cell, especially focusing upon interphase nuclear extra-chromosomal particles (e.g. nucleoli, Cajal bodies, PML bodies, etc.) and on relatively ‘short’ oligonucleosome particles, collectively referred to as ‘biomolecular condensates’ [21–24]. One review [21] argues that ‘multivalent macromolecular interactions’ are the driving force for phase separations. Furthermore, in the final part of this review Discussion, the authors speculate that polynucleosomal phase separation may be important for chromatin structure and function. In a recent publication from our group [28], we proposed that the nucleosome is a *multivalent* structure with histone ‘tails’, intrinsically disordered peptide regions (IDPR) extending from each mononucleosome [45,46]. Given the acknowledged ‘promiscuous’ binding interactions of IDPR [47,48], we proposed an ‘Unstructured Stability’ hypothesis, which postulates that the stability and plasticity of chromatin higher-order structure is a consequence of the collective contributions of numerous weak histone IDPR binding interactions arising from the multivalent nucleosome, analogous to antibody ‘avidity’. However, we cannot claim (although, we suspect) that mitotic chromosome congelation is a form of liquid-liquid phase separation. LLPS is a dynamic process; whereas, congelation has only been studied in HCHO-fixed preparations. Congealed mitotic chromosomes have structural attributes that are consistent with an in vivo ‘condensate’ history. Congealed mitotic chromosomes exhibit variable deformations, segregation (demixing) of ‘normally’ integrated components and a ‘smooth’ epichromatin surface (indicative of ‘surface tension’?). Fission of chromatin ‘droplets’ cannot be confirmed; but evidence that mitotic chromosome arms have ‘fused’ appears compelling. In comparison to the well-studied ‘condensates’ of smaller macromolecular complexes, it seems reasonable to assume that fusion (congelation) of chromosome-sized polynucleosome chains can result in massive and complex macromolecular gels. Indeed, from the perspective of LLPS mechanics [21–24], hyperosmotic cell dehydration might drive mitotic chromosomes to exceed the ‘critical concentration for phase separation’, resulting in a (mitotic) chromatin gel phase with a paucity of chromosome-associated proteins (e.g., Ki67, CTCF, SMC2, RAD 21, HMGs and H1s). The distinctly non-spherical shapes of the congealed gel residues of mitotic chromosome clusters could represent a ‘balance of forces’; i.e., surface tension cohesion versus internal chromatin rigidity. The stability of the congealed state could arise from close-packed multiple interactions of intrinsically disordered peptide regions (i.e., ‘Unstructured Stability’ [28]).

An imperative question is whether chromatin and chromosome congelation can be readily reversed in vivo. Presumably, this would best be attempted when the cells still exhibit high viability, having incurred only a short interval of osmotic pressure dehydration. In this ‘Acute’ phase, the dramatic ‘global’ changes (esp., mitotic chromosome congelation) could be an important structural parameter to search for reversibility. The phase separation of Ki67 combined with the apparent fusion of chromosome arms might be amenable to study by live cell fluorescent microscopy. Similar experiments could involve CTCF, SMC2 and RAD21. These proteins, among others, are critical to mitotic chromosome ‘nested’ looping and contraction [18–20].

Cellular mechanistic information is needed on the polymer properties of chromatin fibers in vivo, including: structural responses to dehydration; to increased ionic and nonionic solute concentrations; to the entropy-driven excluded volume effect and other fluid changes [49,50]. It is of interest that the effects of externally applied pressure on live cells (by compressing with weights on top of coverslips above cells) perturbs nuclear architecture in a way which is similar to the effects of hyperosmotic pressure [51]. These compressive (physical weight) forces induce reversible interphase chromatin condensation. Also of interest, when granulocytic forms of HL-60/S4 migrate thru narrow pores, distorting the nuclear shape, genetic expression is significantly altered [52]. In our view, in vivo phase transition responses of chromatin and chromosomes to external medium conditions are essential to understanding cellular homeostatic mechanisms.

## Materials and methods

### Cell culture

HL-60/S4 (available from ATTC #CRL-3306) and U2OS (ATTC #HTB-96) were cultivated as described earlier [26,41]. Typically, 5 ml of growing HL-60/S4 cells was added to a T-25 flask containing dry sucrose (or NaCl). The flask was oscillated to dissolve the added solutes and returned to the incubator. U2OS cells were grown in 6 well plates and allowed to attach to sterile coverslips. Growth medium was removed by aspiration and replaced with medium made 300 mM sucrose, followed by a return to the incubator.

### Antibodies and immunostaining

The following primary rabbit antibodies were purchased from Abcam: anti-H1.2, ab17677; H1.5, ab18208; HMGB2, ab124670; Ki67, ab15580; H3S10p, ab5176; CTCF, ab128873; SMC2, ab10412; RAD21, ab154769; H1x, ab31972. From Cell Signaling: anti-HMGN1, #5692; HMGN2, #9437. From Sigma-Aldrich: anti-H1.4, H7665. From Millipore: anti-H2A, #07-146. The following mouse antibodies were purchased from Abcam: anti-H3, ab24834; H4, ab31830; H2B, ab52584. From Active Motif: anti-H3, #61475. mAb PL2-6 was a gift from M. Monestier (Temple Univ.), see previous use [26,27]. Secondary antibodies included Invitrogen Alexa 568 goat anti-rabbit IgG, Sigma-Aldrich Atto 488 goat anti-rabbit IgG and Atto 488 goat anti-mouse IgG.

### Confocal microscopy

Most confocal immunostaining was performed on a Leica SP8 microscope followed by deconvolution using AutoQuant X3, employing an adaptive PSF, as described earlier [27].

### STED microscopy

Super-resolution imaging was performed on a home-built STED system described previously [27]. STED images of DAPI were acquired using 405 nm pulsed excitation and 592 nm continuous-wave depletion, while detecting fluorescence in the 515 nm – 565 nm range. This configuration allowed sequential dual-color STED of DAPI in combination with immunostaining with Atto 488 (imaged using 500 nm pulsed excitation) using the ‘long Stokes’ shift’ approach [53] such that both probes are depleted at 592 nm, detected in the same spectral region, and distinguished according to their respective excitation source. STED images were acquired at a scan rate of 2000 lines/s using a pixel size of 20 nm and 100 to 120 line accumulations. Laser power for excitation and depletion was measured at the back focal plane of the objective lens to be ~10 µW and ~160 mW, respectively. Post-acquisition, STED images of DAPI were deconvolved with AutoQuant X3 using a measured STED point spread function obtained by imaging 40 nm fluorescence beads.

### Radial distribution calculations

Prior to calculating the radial distribution (density-density pair correlation) function, each DAPI STED image was masked to segment the interphase nucleus (or mitotic chromosomes) by Gaussian smoothing the image (standard deviation of 5 pixels) and then computing a cluster map using Matlab’s built-in k-means clustering function (with *k* = 2). A grayscale analog of the radial distribution function, $g\left(r\right)$, was computed according to

$$g\left(r\right)=\frac{1}{F\rho }\overset{N}{\underset{k=1}{\sum }}I_{k}f\left(r_{k}\right)/A_{r_{k}}$$

where k runs over all pixels in the (masked) image, F is the sum of all masked signal in the image, ρ is the averge signal density, computed by dividing F by the area of the mask, $I_{k}$ is the intensity at pixel k, $f\left(r_{k}\right)$ is the signal summed in the ring of radius r concentric with pixel k, and $A_{r_{k}}$ is the area of that ring contained within the mask. This weighting by area provides ‘edge-correction’ so that pixels near the boundary of the nucleus (or mitotic chromosomes) do not introduce bias into the calculation. The radial distribution results shown in Figure 4 represent the average ± standard error of (*N)* DAPI STED images analyzed for each condition. To determine the radial position of the ‘peaks’, each curve was locally (i.e. in the immediate vicinity of each ‘peak’) fitted to a quadratic function and the resulting fit parameters were used to calculate the position where the slope of the quadratic function was equaled to zero.

### Gel electrophoresis and immunoblotting

SDS-PAGE was performed on pre-cast 4-12% gradient gels (Expedeon) and stained with InstantBlue. Proteins from the various acid extracts were electrophoretically transferred to 0.45 µm Immobilon-P (Millipore). HRP-labeled secondary antibody reactions were developed with ECL blotting reagents (Amersham) and visualized on a FluorChem imager (ProteinSimple).

### Mass spectroscopy

Protein bands of interest were excised from the gel, cut into small pieces and transferred into a 0.2 mL vial. Gel pieces were washed twice with ABC buffer (100 mM, pH 8.0), twice with ABC buffer/Acetonitrile (1:1) and dried in a vacuum centrifuge. To reduce the disulfide bonds within the proteins, 60 µL of 10 mM DTT in ABC buffer was added to the gel pieces followed by incubation at 56°C for 30 min. Gel pieces were centrifuged, the supernatant removed, and 100 µL acetonitrile was added. After 10 min of incubation, acetonitrile was replaced by 60 µL of 55 mM iodoacetamide in ABC buffer. After 20 min of incubation at room temperature in the dark, the gel pieces were washed with ABC buffer for 5 min and shrunken by adding 100 µL of acetonitrile for 15 min. After removing the supernatant, the gel pieces were again dried in a vacuum centrifuge. The gel pieces were then swollen in 20 µL of 10 mM ammonium bicarbonate buffer containing 0.1 µg of trypsin (Sequencing grade, Promega). For enzymatic digestion, the samples were incubated for 16 h at 37°C. Peptides were thereafter extracted by adding 15 μL of acetonitrile/5% formic acid (2:1) to the gel pieces and incubating at 37°C for 15 min. After collecting the supernatant, the gel extraction was repeated with 10 µL of acetonitrile/5% formic acid (2:1), isopropanol/2% formic acid (1:1), and with acetonitrile (each time incubating at 37°C for 15 min). The supernatants were combined, lyophilized, and stored at −20°C for subsequent analysis.

Tryptic digests were analyzed using an UltiMate 3000 RSCLnano-HPLC system coupled to a Q Exactive HF mass spectrometer (both, Thermo Scientific, Bremen, Germany) equipped with a Nanospray Flex ionization source. The peptides were separated on a homemade fritless fused-silica micro-capillary column (100 µm i.d. x 280 µm o.d. x 20 cm length) packed with 2.4 µm reversed-phase C18 material. Solvents for HPLC were 0.1% formic acid (solvent A) and 0.1% formic acid in 85% acetonitrile (solvent B). The gradient profile was as follows: 0–4 min, 4% B; 4–57 min, 4-35% B; 57–62 min, 35-100% B, and 62–67 min, 100% B. The flow rate was 300 nl/min.

The Q Exacitve HF mass spectrometer was operating in the data dependent mode selecting the top 20 most abundant isotope patterns with charge >1 from the survey scan with an isolation window of 1.6 mass-to-charge ratio (m/z). Survey full scan MS spectra were acquired from 300 to 1750 m/z at a resolution of 60,000 with a maximum injection time (IT) of 120 ms, and automatic gain control (AGC) target 1e6. The selected isotope patterns were fragmented by higher-energy collisional dissociation with normalized collision energy of 28 at a resolution of 30,000 with a maximum IT of 120 ms, and AGC target 5e5.

Data Analysis was performed using Proteome Discoverer 2.1 (Thermo Scientific) with search engine Sequest. The raw files were searched against the uniprot homo sapiens database (UP000005640_9606, 20,937 sequences). Precursor and fragment mass tolerance was set to 10 ppm and 0.02 Da, respectively, and up to two missed cleavages were allowed. Carbamidomethylation of cysteine was set as static modification and oxidation of methionine as variable modification. Acetylation, methionine-loss, and methionine-loss plus acetylation were set as N-terminal dynamic modification of proteins. Peptide identifications were filtered at 1% false discovery rate.

### Cell viability and mitochondrial polarization measurements

Measurements were made on a Guava easyCyte single-cell analysis system (EMD Millipore Corp.) Viability was determined using Guava ViaCount Reagent (Luminex) 4000–0040, following the manufacturer’s instructions. Mitochondrial membrane polarization was determined employing a Muse MitoPotential Kit (EMD Millipore Corp.) MCH100110-2, following the manufacturer’s instructions. Cells were incubated for various lengths of time (0, 30 min, 1, 2 and 3 hours) in RPMI-1640 medium made 300 mM sucrose and all analyzed at the same time.

### Cell sizing

Estimates of cell sizes (i.e., average diameter and spherical volume) for HL-60/S4 cells treated with 300 mM sucrose or 150 mM NaCl were obtained with a Multisizer 4 (Beckman Coulter Life Sciences). Cells were incubated for 30 and 60 min in RPMI medium plus 300 mM sucrose or 150 mM NaCl at 37°C and diluted to ~1-2x10^4^ cells/ml in Isotone II diluent plus sucrose or NaCl at RT, just prior to diameter analysis. Cell volumes were estimated by assuming that the cells are spheres (undifferentiated HL-60/S4 cells grow in suspension).

### Cell cycle analyses

Rapidly growing HL-60/S4 cells were incubated in medium plus 300 mM sucrose for 15 and 30 minutes and for 1 hour at 37°C. Control and sucrose-treated cells were centrifuged at 300xg for 6 minutes at 10°C and washed once with cold PBS or PBS plus 300 mM sucrose. To 0.5 ml concentrated cell suspension, 4.5 ml of cold 70% ethanol was added. The cell suspensions were stored in the cold (4-10°C) until analysis 4 days later. Propidium Iodide was added to the fixed cell suspensions just before analytical fluorescence cytometry, which was performed with a Miltenyi Biotec MacsQuant 10.
