# Supplementary material for: Hyperosmotic stress: in situ chromatin phase separation
Source: Nucleus. 2020 Jan 10;11(1):1–18. doi: 10.1080/19491034.2019.1710321 (PMC6973338; doi:10.1080/19491034.2019.1710321)
Supplement: Supplemental Material [file kncl-11-01-1710321-s001.zip › Supplementary information/2019-12-13 Table S1.docx]

**TABLE S1. MASS SPECTROSCOPIC AND IMMUNOBLOT RESULTS FOR HISTONES AND HMG PROTEINS IN PCA AND H_2_SO_4_ EXTRACTS**

| **PCA**  **Band Name** | **MW (kDa)**  **apparent** | **Protein Name**  **0 mM Sucrose** | **Protein Name**  **300 mM Sucrose** | **MW (kDa)**  **calculated** |
| --- | --- | --- | --- | --- |
| HMG | ~18 | H1.5 | nt | 22.6 |
|  |  | HMGA1 | nt | 11.7 |
|  |  | HMGN1 | nt | 10.7 |
|  |  | (HMGN2) | nt | 9.4 |
|  |  |  |  |  |
| H1 | ~33 | H1.2 | nt | 21.4 |
|  |  | H1.4 | nt | 21.9 |
|  |  | H1.5 | nt | 22.6 |
|  |  | H1x | nt | 22.5 |
|  |  | HMGA1 | nt | 11.7 |
|  |  | HMGB1 | nt | 24.9 |
|  |  | HMGB2 | nt | 24 |
|  |  | HMGN1 | nt | 10.7 |
|  |  | (HMGN2) | nt | 9.4 |
|  |  |  |  |  |
| (H1)_2_ | ~68 | H1.2 | H1.2 | 21.4 |
|  |  | H1.4 | -- | 21.9 |
|  |  | H1.5 | H1.5 | 22.6 |
|  |  | H1x | -- | 22.5 |
|  |  | HMGB1 | -- | 24.9 |
|  |  | HMGB2 | -- | 24 |
|  |  | (HMGN1) | -- | 10.7 |
|  |  | HMGN2 | -- | 9.4 |
|  |  | HMGN5 | -- | 31.5 |
|  |  |  |  |  |
| **H_2_SO_4_**  **Band Name** |  |  |  |  |
| Pol1 | ~48 | H1.2 | H1.2 | 21.4 |
|  |  | H1.4 | H1.4 | 21.9 |
|  |  | H1.5 | H1.5 | 22.6 |
|  |  | H1x | -- | 22.5 |
|  |  | HMGA1 | -- | 11.7 |
|  |  | HMGB1 | -- | 24.9 |
|  |  | HMGB2 | --- | 24 |
|  |  | HMGB3 | -- | 23 |
|  |  | HMGN1 | -- | 10.7 |
|  |  | (HMGN2) | -- | 9.4 |
|  |  | H2A | -- | 14.1 |
|  |  | Macro-H2A.1 | -- | 39.6 |
|  |  | H2A.Z | -- | 13.5 |
|  |  | -- | H2AX | 15.1 |
|  |  | H2B | H2B | 13.9 |
|  |  | H3 | -- | 15.4 |
|  |  | H3.1 | H3.1 | 15.4 |
|  |  | H4 | -- | 11.4 |
|  |  |  |  |  |
| Pol2 | ~65 | H1.2 | H1.2 | 21.4 |
|  |  | H1.4 | H1.4 | 21.9 |
|  |  | H1.5 | H1.5 | 22.6 |
|  |  | H1x | -- | 22.5 |
|  |  | HMGA1 | -- | 11.7 |
|  |  | HMGB1 | -- | 24.9 |
|  |  | HMGB2 | -- | 24 |
|  |  | HMGB3 | HMGB3 | 23 |
|  |  | HMGN1 | -- | 10.7 |
|  |  | HMGN2 | -- | 9.4 |
|  |  | HMGN5 | HMGN5 | 31.5 |
|  |  | H2A | -- | 14.1 |
|  |  | H2A.Z | H2A.Z | 13.5 |
|  |  | H2B | H2B | 13.9 |
|  |  | H3 | -- | 15.4 |
|  |  | H3.1 | H3.1 | 15.4 |
|  |  | H4 | -- | 11.4 |
|  |  |  |  |  |
| Pol3 | ~80 | H1.2 | H1.2 | 21.4 |
|  |  | H1.4 | H1.4 | 21.9 |
|  |  | H1.5 | H1.5 | 22.6 |
|  |  | HMGB1 | HMGB1 | 24.9 |
|  |  | HMGB2 | HMGB2 | 24 |
|  |  | HMGB3 | -- | 23 |
|  |  | HMGN1 | -- | 10.7 |
|  |  | HMGN2 | -- | 9.4 |
|  |  | H2A | -- | 14.1 |
|  |  | H2A.Z | H2A.Z | 13.5 |
|  |  | H2B | H2B | 13.9 |
|  |  | H3 | -- | 15.4 |
|  |  | H3.1 | H3.1 | 15.4 |
|  |  | H4 | H4 | 11.4 |
