# Supplementary material for: Hyperosmotic stress: in situ chromatin phase separation
Source: Nucleus. 2020 Jan 10;11(1):1–18. doi: 10.1080/19491034.2019.1710321 (PMC6973338; doi:10.1080/19491034.2019.1710321)
Supplement: Supplemental Material [file kncl-11-01-1710321-s001.zip › Supplementary information/Supplementary Figures.docx]

Supplementary Figure S1. Confocal immunostaining of U2OS cells fixed in the absence/presence of 300 mM sucrose. Stained with mAb PL2-6 (anti-epichromatin, red) and DAPI (cyan); (**a,b**), interphase nuclei; (**c,d**), mitotic chromosomes. (**a,c**), 0 mM sucrose; (**b,d**), 300 mM sucrose. Magnification bar, 10 µm.

Supplementary Figure S2. STED and confocal imaging of mitotic chromosome clusters from HL-60/S4 cells fixed in the absence/presence of 100 and 200 mM sucrose, stained only with rabbit anti-Ki67. (**a,b,c**), STED; (**d,e,f**), confocal imaging. (**a,d**), 0 mM sucrose. (**b,e**), 100 mM sucrose. (**c,f**), 200 mM sucrose. Ki67 staining is shown in an inverse gray scale, where “white” is the most intense staining. Magnification bar, 10 µm.

Supplementary Table S1. Mass spectroscopy (MS) and immunoblot (IB) results for histones and HMG proteins in PCA and H_2_SO_4_ extracts from HL-60/S4 cells, following DSS crosslinking (1 mM DSS, 5 min) in the presence of 0 or 300 mM sucrose. For MS, see Fig. 7**b**, gel slices of PCA extracts were collected from three bands crosslinked in 0 mM sucrose [HMG, H1 and (H1)_2_] and from one band crosslinked in 300 mM sucrose [(H1)_2_]. In addition, gel slices of H_2_SO_4_ extracts were collected from three bands crosslinked in 0 mM sucrose [Pol1, Pol2 and Pol3] and from three bands crosslinked in 300 mM sucrose [Pol1, Pol2 and Pol3]. “Convenience Names” for these bands are listed in column 1. The apparent molecular weights of the proteins in each band are indicated in column 2. Common names for the proteins detected by MS and/or IB are shown in column 3. A parenthesis around a protein name in column 3, indicates that the protein was not detected in the PCA extract from cells crosslinked by DSS in 0 mM sucrose, but was detected by IB of the PCA extract. Column 4 lists the names of proteins detected in PCA or H_2_SO_4_ extracts of cells crosslinked in 300 mM sucrose. Column 5 presents the protein molecular weights calculated from their amino acid content. Note that for essentially all of these proteins, the calculated MW is less than the apparent MW, reflecting their high % of basic residues (if monomers) and/or their crosslinking to other extracted proteins. Notations: “nt”, not analyzed by MS; “-“, not detected by MS; “⚫️”, strong-medium IB reaction; “🔘”, weak-trace IB reaction. The relative band intensities were judged from several experiments and several exposures for each experiment. The antibody against histone H3 does not distinguish between H3 and H3.1; however, MS does make that distinction. In one MS run of “HMG”, inner histones were detected. The suspicion that this represented a contamination by an adjacent lane of inner histones was confirmed; therefore, these proteins are not listed. This table is limited to histones and HMG proteins with an “accumulation” of greater than/equal to E+06.
