# Supplementary figures and images for: Hyperosmotic stress: in situ chromatin phase separation
Source: Nucleus. 2020 Jan 10;11(1):1–18. doi: 10.1080/19491034.2019.1710321 (PMC6973338; doi:10.1080/19491034.2019.1710321)

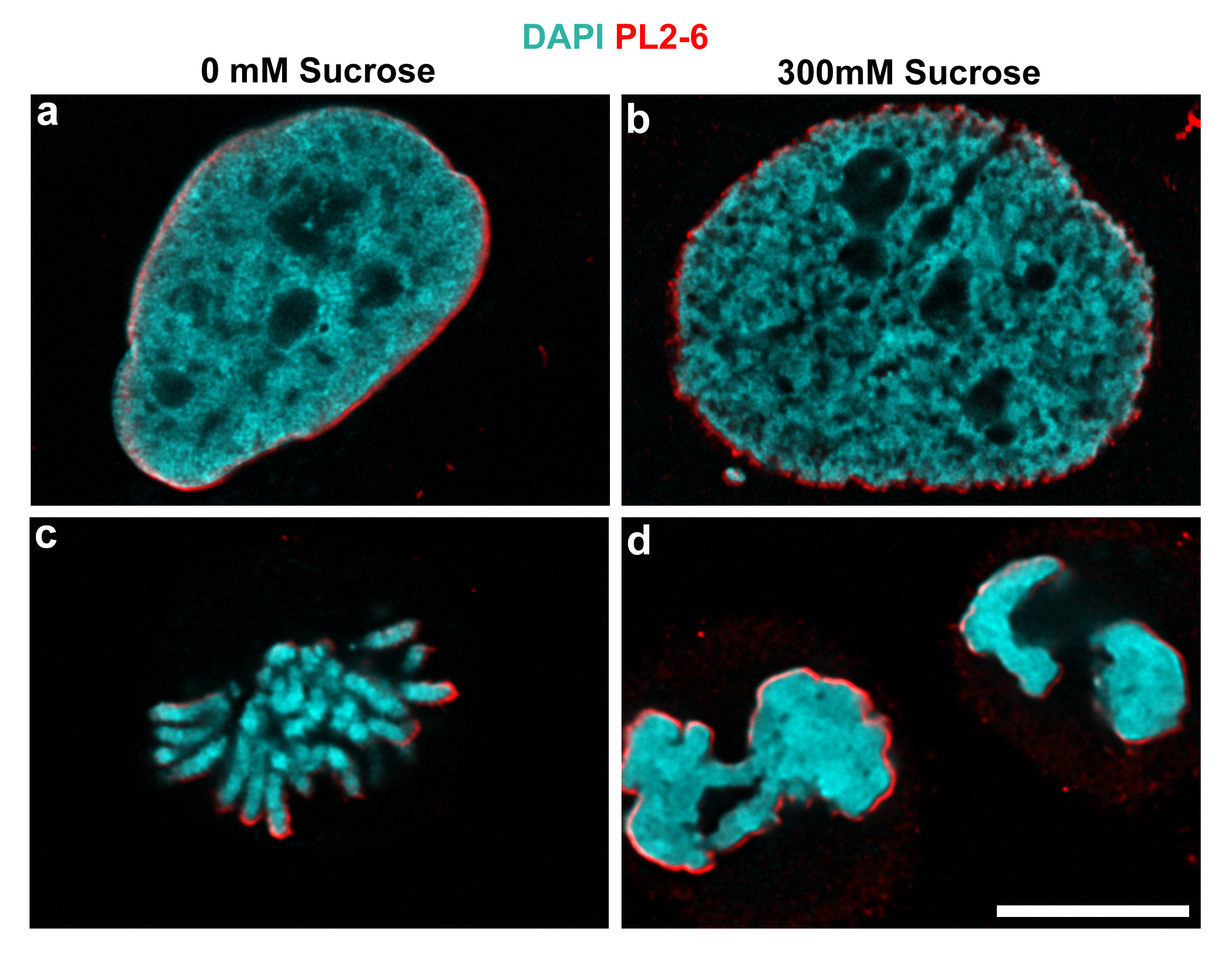

Supplement: Supplemental Material [file kncl-11-01-1710321-s001.zip › Supplementary information/Figure S1.tif]

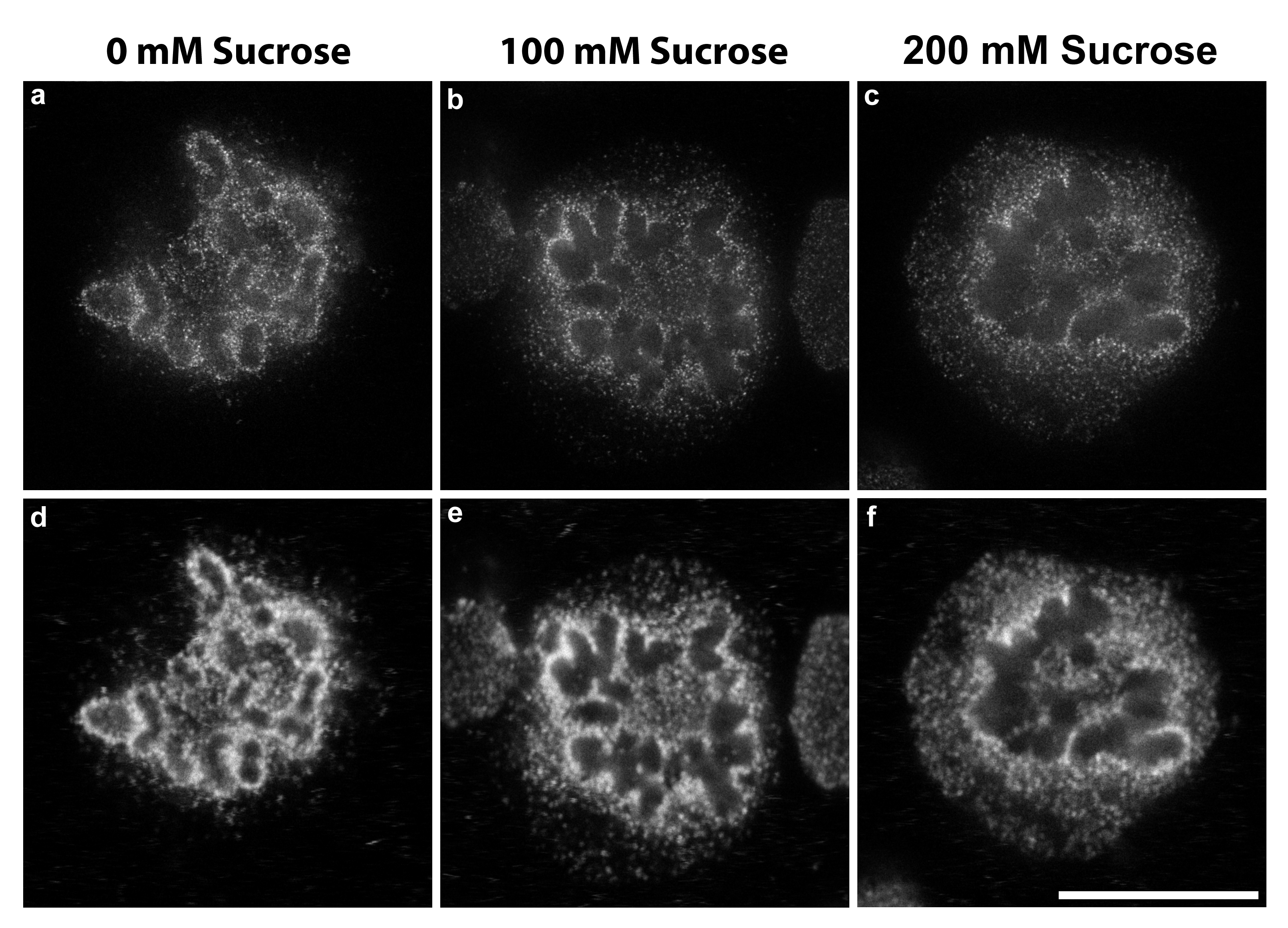

Supplement: Supplemental Material [file kncl-11-01-1710321-s001.zip › Supplementary information/Figure S2.tif]
